# Excitonic splittings in molecular dimers: why static *ab initio* calculations cannot match them

**DOI:** 10.1039/c5sc02546j

**Published:** 2015-08-26

**Authors:** Philipp Ottiger, Horst Köppel, Samuel Leutwyler

**Affiliations:** a Dept. für Chemie und Biochemie , Freiestrasse 3 , CH-3012 Bern , Switzerland . Email: leutwyler@iac.unibe.ch ; Fax: +41 31 631 3940 ; Tel: +41 31 631 4479; b Physikalisch-Chemisches Institut , Universität Heidelberg , Im Neuenheimer Feld 229 , D-69120 Heidelberg , Germany

## Abstract

After decades of research on molecular excitons, only few molecular dimers are available on which exciton and vibronic coupling theories can be rigorously tested. In centrosymmetric H-bonded dimers consisting of identical (hetero)aromatic chromophores, the monomer electronic transition dipole moment vectors subtract or add, yielding S_0_ → S_1_ and S_0_ → S_2_ transitions that are symmetry-forbidden or -allowed, respectively. Symmetry breaking by ^12^C/^13^C or H/D isotopic substitution renders the forbidden transition weakly allowed. The excitonic coupling (Davydov splitting) can then be measured between the S_0_ → S_1_ and S_0_ → S_2_ vibrationless bands. We discuss the mass-specific excitonic spectra of five H-bonded dimers that are supersonically cooled to a few K and investigated using two-color resonant two-photon ionization spectroscopy. The excitonic splittings *Δ*_calc_ predicted by *ab initio* methods are 5–25 times larger than the experimental excitonic splittings *Δ*_exp_. The purely electronic *ab initio* splittings need to be reduced (“quenched”), reflecting the coupling of the electronic transition to the optically active vibrations of the monomers. The so-called quenching factors *Γ* < 1 can be determined from experiment (*Γ*_exp_) and/or calculation (*Γ*_calc_). The vibronically quenched splittings *Γ*·*Δ*_calc_ are found to nicely reproduce the experimental exciton splittings.

## Abstract 2

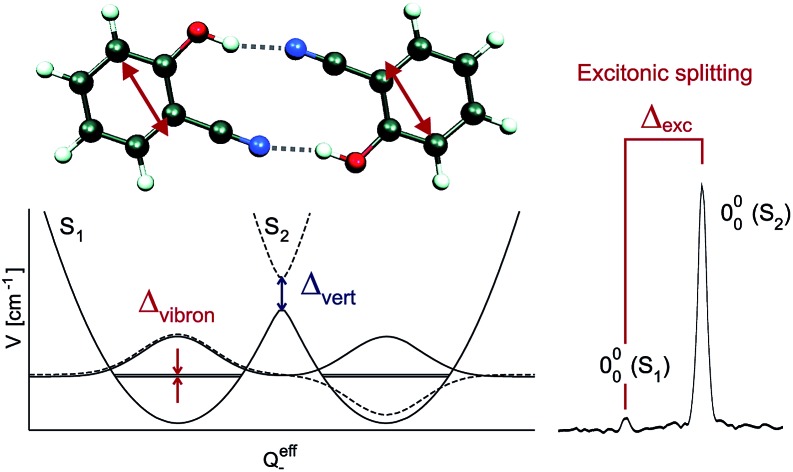
We show experimentally that excitonic splittings in symmetric dimers calculated *ab initio* are 5–25 times too large, and explain why using vibronic coupling theory.

## 1 Introduction

Molecular excitons are collective excited states that are important for the function of a wide range of biological and chemical multichromophoric systems. These involve weakly interacting ultraviolet (UV) or visible chromophores with distinctly non-additive excited-state properties. Molecular excitons occur in molecular crystals, conjugated polymers with aromatic side groups, photosynthetic light-harvesting antenna systems, photosynthetic reaction centers and nucleic acids.^1^–^11^ In all of these systems, the excitonic interactions have a significant impact on the electronic structure and functions. Depending on the strength of the intermolecular electronic interactions one observes time-independent spectroscopic phenomena such as line splittings or band splittings, or alternatively time-dependent photophysical effects such as long-range energy transfer, as succinctly summarized by Förster,^12^ see Table 1.

**Table 1:** Phenomenological classification of excitonic interactions^12^

Coupling Strength	Quant.-mech. Treatment	Electronic States	Experimental Effects
Strong	Stationary	Delocalized	Separated band systems
Weak	Stationary	Partially local.	Band splittings, intensities
Very weak	Time-depdt.	Localized	Excitation transfer (FRET)

Excitonic coupling in molecular dimers and larger aggregates has been theoretically studied since the late 1950s,^13^–^24^ but only few experimental investigations have rigorously tested the predictions of exciton coupling theories. In the following, we discuss vibronically resolved ultraviolet UV spectra of symmetric molecular homo-dimers that are formed, rotationally and vibrationally cooled to a few degrees K and isolated in supersonic jets. These dimers consist of identical chromophores denoted A and B. If the A·B dimer structure is centrosymmetric (*C*_*i*_ or *C*_2h_) and the monomers are exchanged by an inversion *î*, the electronic transition-dipole moment vectors of the local S_0_ → S_1_ transitions of A and B combine exactly parallel or antiparallel. Of the resulting dimer S_0_ → S_1_ and S_0_ → S_2_ transitions, one is electric-dipole forbidden, while the other is symmetry-allowed. However, minimal symmetry perturbations such as ^12^C/^13^C- or H/D isotopic substitution lift the symmetry restrictions of the forbidden transition sufficiently to render both transitions allowed.^25^–^30^ The excitonic interaction (coupling) between A and B can then be measured as the Davydov splitting energy *Δ*_exc_ = 2*V*_AB_ between the S_0_ → S_1_ and S_0_ → S_2_ vibrationless transitions, where *V*_AB_ is the excitonic coupling matrix element.^16^

By combining laser vibronic spectroscopy with mass-specific (*i.e.* H/D or ^12^C/^13^C isotope-specific) detection, the excitonic splittings of different isotopic species can be measured. These provide strict benchmarks for the predictions of exciton and vibronic coupling theories. Current improvements of these theories^31^–^34^ can be tested and will provide a deeper understanding of excitonic interactions in dimers and larger multichromophoric systems.

In a symmetric molecular dimer A·B, the monomer S_0_ → S_1_ excitations are simultaneously subject to two interactions, (i) the exchange of the electronic excitation energy that tends to distribute the excitation uniformly over A and B and (ii) the electronic–vibrational coupling which tends to localize the excitation on either one of the monomers by linking it to a vibrational displacement. The “weak” and “strong” dimer vibronic coupling cases were defined in the 1960s in terms of the relative size of the excitonic and vibronic couplings.^13^–^15^,^17^ A first model treatment of linear vibronic coupling of two electronic states in a symmetric dimer was undertaken by Witkowski and Moffitt^15^ and by Fulton and Gouterman^18^,^19^ 50 years ago; these concepts were later extended by other workers.^20^–^24^

## 2 Excitonic splitting: basic features

**Fig. 1:**
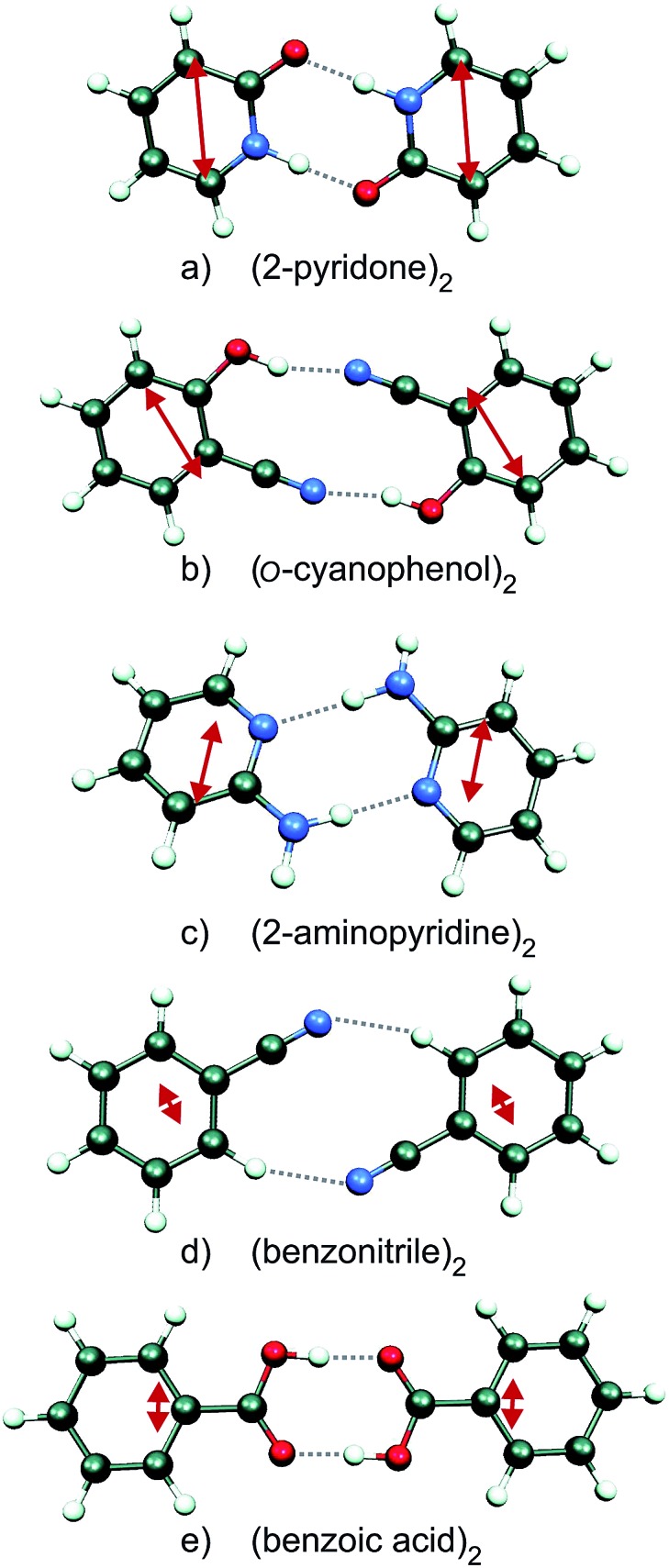
Ground state geometries of the H-bonded dimers (2-pyridone)_2_, (*o*-cyanophenol)_2_, (2-aminopyridine)_2_, (benzonitrile)_2_ and (benzoic acid)_2_ (CC2/aug-cc-pVTZ calculations). The monomer transition-dipole moment vectors are indicated as red double-headed arrows.

We have investigated the *C*_2h_- or *C*_2_-symmetric doubly H-bonded homodimers (2-pyridone)_2_, (*o*-cyanophenol)_2_, (2-aminopyridine)_2_, (benzonitrile)_2_ and (benzoic acid)_2_ shown in Fig. 1. The close-lying H-bond donor/acceptor groups of the monomers lead to rigid self-dimers with well-defined distances and orientations between the monomer electronic transition dipole moment vectors ***μ⃑***_A_, ***μ⃑***_B_. For (2-pyridone)_2_, (benzonitrile)_2_ and (benzoic acid)_2_, the ground- and excited-state gas-phase structures have been determined by laser high-resolution spectroscopy.^35^–^38^ The infrared and UV spectra of the *o*-cyanophenol dimer, the *m*-cyanophenol dimer and the mixed *o*-cyanophenol-*m*-cyanophenol dimer have been investigated at vibronic resolution by Lahmani, Zehnacker and co-workers,^39^,^40^ and similarly for the anthranilic acid (2-amino-benzoic acid) dimer by Levy, Zwier and co-workers.^41^ We also note several theoretical and spectroscopic studies of the stacked anisole dimer,^42^–^44^ although stacked dimers are outside the scope of this short review.

**Fig. 2:**
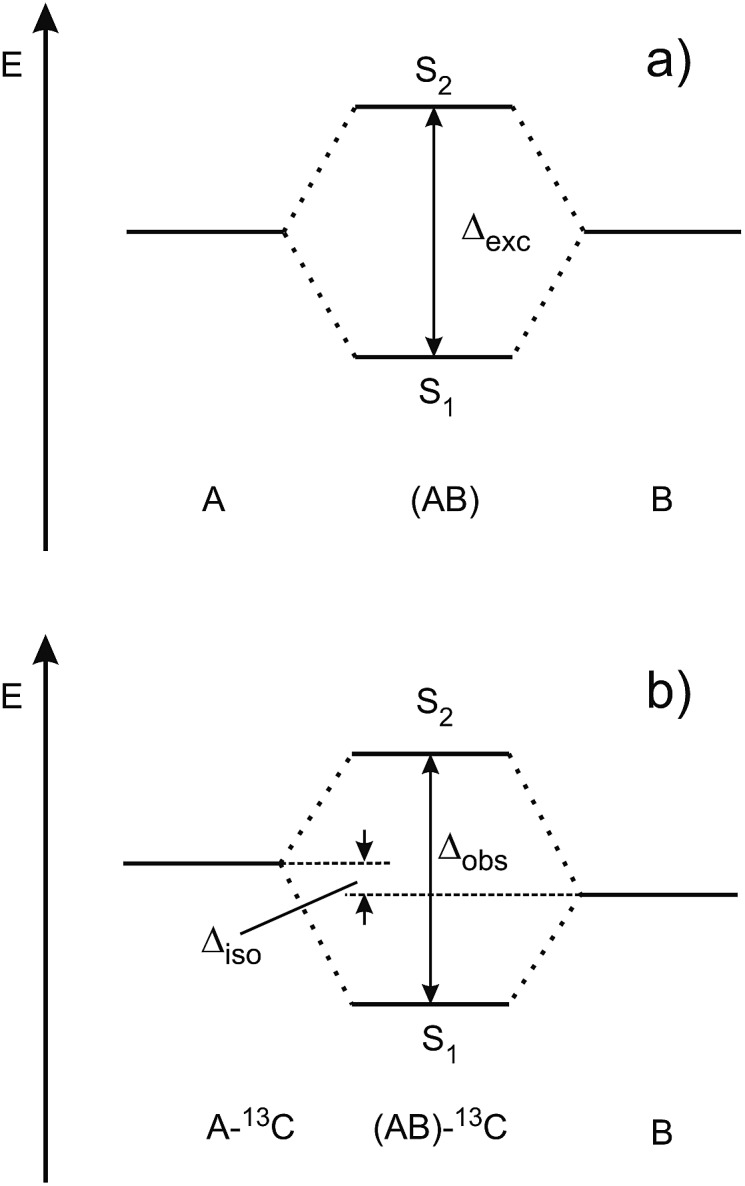
Schematic view of (a) the excitonic splitting in a symmetric dimer consisting of identical chromophores A and B, (b) the S_1_/S_2_ state splitting in a symmetry-broken dimer that is isotopically substituted in chromophore A.

Since the S_0_ → S_1_ excitation of the monomers is in-plane ππ*, the excitonic interaction in the dimer is dominated by the respective transition-dipole moment vectors, which combine in parallel or antiparallel manner, giving rise to the S_0_ → S_1_ and S_0_ → S_2_ excitations of the dimer, see Fig. 1 and 2. In the *C*_2h_ dimers, one transition is A_g_ → A_g_ and is strictly electric-dipole forbidden, while the other transition is A_g_ → B_u_, is allowed and is also experimentally observed. However, even a single ^12^C/^13^C- or H/D-isotopic substitution lead to sufficiently large deviations from inversion-symmetry as to render the A_g_ → A_g_ transition weakly allowed and observable. Mass-selective detection of the ^13^C-isotopomer spectra of the dimers in Fig. 1, which exhibit 10–15% of the intensity of the all ^12^C-isotopomers due to the natural ^13^C content, in combination with UV/UV holeburning techniques allow to record isotopomer-specific cold gas-phase absorption spectra.

**Fig. 3:**
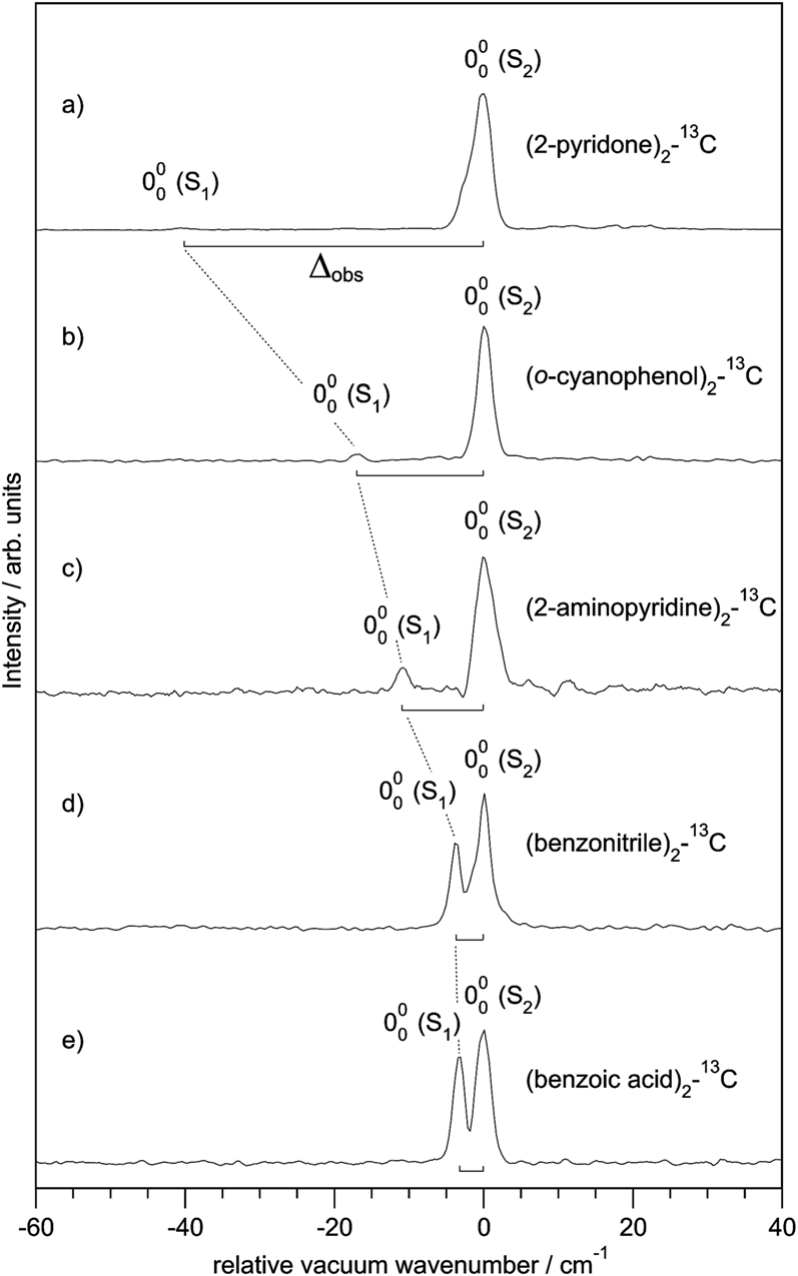
Experimentally observed splittings *Δ*_obs_ between the S_1_ and S_2_ electronic origins of the ^13^C-isotopomers of the doubly H-bonded dimers in Fig. 1.

Fig. 3 shows the spectroscopically observed splittings between the S_0_ → S_1_ and S_0_ → S_2_ transitions of the ^13^C-dimers in Fig. 1. As indicated in Fig. 2, the ^12^C/^13^C substitution renders the monomers unequal, hence an additional isotopic splitting *Δ*_iso_ arises from the isotope-induced differences of zero-point vibrational energies of the S_0_ and S_1_ states of the monomer. This results in small changes of the S_0_ → S_1_ excitation energies of the chromophores in the ^13^C-dimer. As a consequence the observed S_1_/S_2_ splitting *Δ*_obs_ is slightly larger than the purely excitonic splitting, *cf.*Fig. 2(b). In second order perturbation theory, the ^12^C/^13^C or H/D isotopic shift *Δ*_iso_ and the excitonic splitting *Δ*_exc_ combine as1
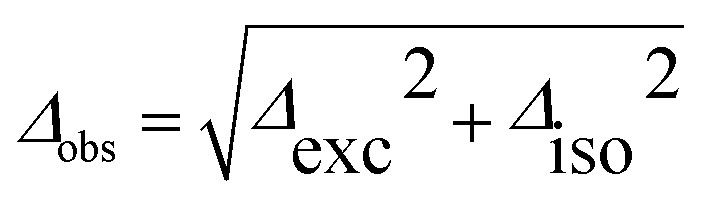



Thus the experimentally observed splittings of the singly ^13^C- or D-substituted dimers are upper limits to *Δ*_exc_. Given a sufficiently large set of isotopomers with different *Δ*_iso_ contributions, the purely excitonic interaction *Δ*_exc_ can then be determined to within 0.5–1 cm^–1^.^26^–^30^

## 3 Experimental techniques

The dimers in Fig. 1 were produced in pulsed supersonic jets using neon carrier gas, resulting in isolated and vibrationally cold (*T*_vib_ ∼ 5 K) complexes.^26^,^28^–^30^ The skimmed molecular beam was spatially and temporally overlapped with an excitation and an ionization laser in the source of a time-of-flight mass spectrometer. By scanning the excitation laser wavelength, the dimers were excited from their S_0_ vibrational ground state to the vibrational levels of the electronic excited state and from there subsequently ionized by the ionization laser at fixed wavelength, according to a two-color resonant two-photon ionization scheme (2C-R2PI). Recording the ion current at different mass channels in dependence of the excitation wavelength yields mass selective absorption spectra of the complexes. The use of UV/UV depletion and holeburning techniques, where the ground state population of a specific isomer is depleted with an additional laser that precedes the excitation laser temporally, allows to record spectra that are both mass and isomer specific.^25^–^30^

## 4 Experimental *vs.* calculated splittings

A number of excited-state quantum-chemical investigations have studied the S_1_, S_2_ and higher S_*n*_ state energies of symmetric molecular dimers. Many of these have focused on face-to-face stacked dimers such as (benzene)_2_,^45^ stacked nucleobases,^46^–^50^ and the anisole dimer.^42^ Our calculations of the excitonic splittings of the H-bonded dimers in Fig. 1 using *ab initio* methods at the CC2/aug-cc-pVTZ level at the symmetric ground-state geometries gave vertical excitonic splittings *Δ*_vert_ between 1100 cm^–1^ and 10 cm^–1^, see column 2 of Table 2. Very similar splitting energies are obtained by calculating the transition-dipole ↔ transition-dipole interaction between the CC2/aug-cc-pVTZ calculated transition-dipole moments of the two monomers (column 3 of Table 2).^12^,^16^ In contrast, the corresponding observed S_1_/S_2_ splittings of the ^13^C-isotopomers *Δ*_obs_ are between 44 cm^–1^ and 0.9 cm^–1^ or 5–25 times smaller (column 5 of Table 2).^26^–^30^ In case of (2-pyridone)_2_, (*o*-cyanophenol)_2_ and (2-aminopyridine)_2_, the contribution of the ^13^C isotopic shift *Δ*_iso_ to the observed splitting is negligible, thus for these dimers *Δ*_obs_ = *Δ*_exp_, see columns 4 and 5 of Table 2.^26^,^28^ In contrast, for (benzonitrile)_2_ and (benzoic acid)_2_, the contribution of *Δ*_iso_ is non-negligible and the excitonic splitting *Δ*_exp_ was deduced from experimental data on several isotopomers.^29^,^30^

**Table 2:** *Ab initio* and dipole–dipole model calculated excitonic splittings of H-bonded dimers, compared to observed S_1_/S_2_ splittings and experimental excitonic splittings *Δ*_exp_ (in cm^–1^)

	*Δ*_vert_	Dipole–dipole	*Δ*_obs_ (Fig. 3)	*Δ*_exp_
(2-Pyridone)_2_	1125	745	43.5	43.5
(*o*-Cyanophenol)_2_	309	299	16.4	16.4
(2-Aminopyridine)_2_	416	362	11.5	11.5
(Benzonitrile)_2_	10	14	3.9	2.1
(Benzoic acid)_2_	11	22	3.4	0.9

All excited-state *ab initio* calculations of the S_1_–S_2_ energy gap, be they vertical or adiabatic, yield excitonic splittings that are systematically larger than the experimental ones. This is not due to an insufficiency of the calculations, but arises from the degeneracy of the two electronic states in symmetric homodimers (or near-degeneracy in the isotopically substituted dimers). In this situation, the Born–Oppenheimer (frozen-nuclei) approximation used in the *ab initio* calculations leads to *electronic* splittings that cannot reproduce the experimental ones. The latter are vibronic (vibrational–electronic) quantities that include the effects of all 3*N* – 6 intra- and intermolecular vibrations of the dimer on the excitonic interaction between the two electronic states. This vibronic coupling significantly reduces or quenches the gap between the two lowest vibronic states, relative to the energy gap between the Born–Oppenheimer calculated S_1_/S_2_ state energies.^18^–^24^,^28^ After explicitly taking this quenching by the vibronic coupling into account, correlated excited-state wave function methods as CC2 are sufficient to obtain accurate results for H-bonded dimers.

## 5 Linear vibronic coupling model for excitonic systems

### 5.1 Hamiltonian and potential energy surfaces

A model for the excitonic coupling of two electronically excited states coupled to a single vibrational mode was introduced by Fulton and Gouterman (FG)^18^,^19^ and numerically solved in 1964.^19^ The dimer electronic ground state is written as a Hartree product of wave functions *φ*_0_ on A and B

2 *Ψ*_0_ = *φ*A0·*φ*B0

Electronic excitation of A or B results in the excited electronic states (where *q* and *Q* refer to electronic and nuclear coordinates)

3 *Ψ*Aexc(*q*; *Q*_0_) = *φ*Aexc(*q*^A^; *Q*^A^)·*φ*B0(*q*^B^; *Q*^B^)

4 *Ψ*Bexc(*q*; *Q*_0_) = *φ*A0(*q*^A^; *Q*^A^)·*φ*Bexc(*q*^B^; *Q*^B^)

Since the Born–Oppenheimer approximation is no longer applicable due to the degeneracy of these excited states, they used as a basis for the vibronic wave function

5 *Ψ*(*q*; *Q*_0_) = *α*(*Q*)·*Ψ*Aexc(*q*; *Q*_0_) + *β*(*Q*)·*Ψ*Bexc(*q*; *Q*_0_)

The parameters *α*, *β* depend on the vibrational coordinate *Q* and are determined by solving the vibronic Hamiltonian (6) where *V*_AB_ represents the excitonic interaction between A and B:6
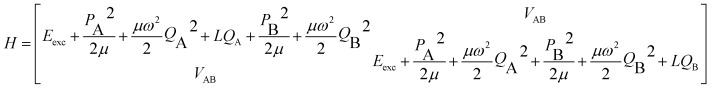



The FG model considers one intramolecular vibration per monomer, which is assumed to be totally-symmetric in the monomer point group. It is represented by a harmonic-oscillator potential 
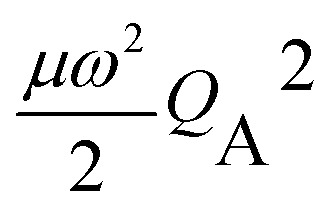
 (*i* = A, B), for which only linear coupling occurs upon electronic excitation, that is, with a horizontal shift *LQ*_*i*_ along the coordinate *Q*_*i*_, but with no change of the reduced mass *μ* or frequency *ω*. For derivatives of benzene, an intramolecular vibration that typically shows considerable vibronic coupling is the in-plane (*a*′) deformation vibration *ν*_6*a*_, as discussed for 2-aminopyridine.^26^ The diagonals of eqn (6) are then transformed to a symmetrized vibrational basis corresponding to the symmetric and antisymmetric combinations of this monomer vibration.

**Fig. 4:**
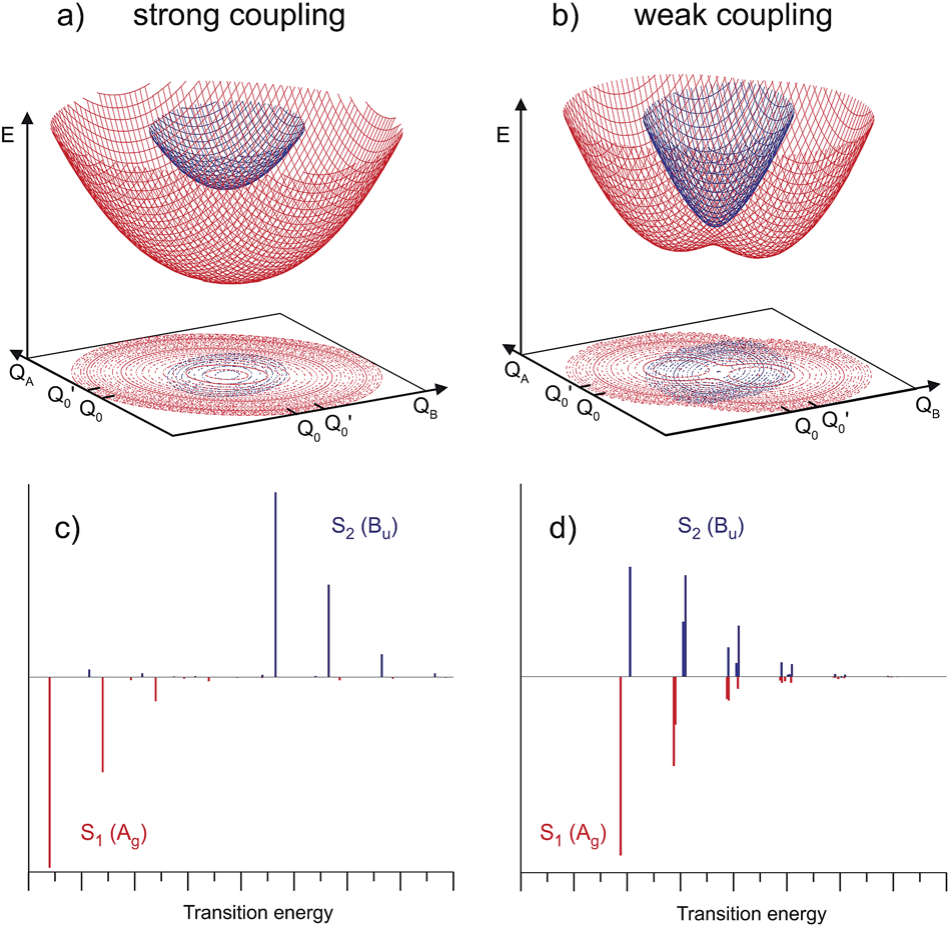
Two-dimensional potential energy surfaces of the S_1_ (red) and S_2_ (blue) states of a symmetric self-dimer, plotted as a function of the intramolecular vibrational coordinates *Q*_A_ and *Q*_B_. (a) Strong coupling case, (b) weak coupling case. The calculated vibronic band patterns are shown as stickplots in (c and d), with negative band intensities (red) for A_g_ vibronic transitions and positive (blue) intensities for the B_u_ vibronic transitions.

Depending on the strength of the excitonic interaction *V*_AB_ between the monomers and the strength of the coupling *LQ*_*i*_ to the vibrational mode, the dimer can be classified as strong- or weak-coupling case.^13^–^15^,^17^ The corresponding potentials and resulting vibronic spectra are shown in Fig. 4. Note that in the use of FG, the coupling strength refers to the strength of the electronic interaction, not to the coupling to vibrational modes. In the case of electronic (excitonic) coupling that is strong relative to the vibrational coupling 
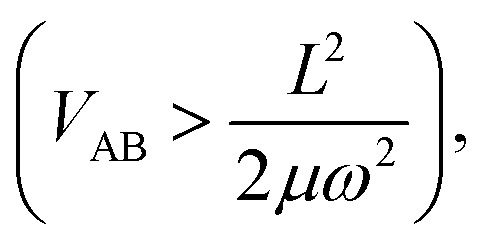
 the S_1_ and S_2_ states of the complex remain largely independent, resulting in well-separated band systems for both states, see Table 1, line 1. Note that the frequency of the antisymmetric vibration in the dimer is lower than *ω* in the lower (red-shifted) state and higher than *ω* in the higher-energy (blue-shifted) state. In case of weak excitonic coupling, the S_1_ and S_2_ states interact considerably, resulting in a double-minimum potential along the antisymmetric direction in the S_1_ state, see Fig. 4(b). The vibronic band systems of the two S_1_ and S_2_ states overlap strongly, giving rise to band splittings and band intensity effects, *cf.*Table 1, line 2.

The excitonic coupling of all the H-bonded molecular dimers in Fig. 1 correspond to the weak-coupling case. For the intramolecular vibrations, experimental data confirms the theoretical prediction of band splitting. Interestingly, band splittings are also observed for the intermolecular vibrational modes of the dimer, resulting in complex band patterns.^25^–^33^ Although the FG model is based on the treatment of intramolecular vibrations, it could be parameterized to reproduce the band splittings and patterns resulting from antisymmetric intermolecular vibrations.^26^,^51^ This, however, is a purely phenomenological approach; recent multimode vibronic coupling^52^ calculations including both intra- and inter molecular vibrational modes on an equal footing are able to reproduce both the inter- and intramolecular vibronic band patterns for (*o*-cyanophenol)_2_.^33^

### 5.2 Vibrational quenching

In weak-coupling dimers, the vibronic coupling not only leads to complicated vibronic band patterns, but also to a considerable reduction of the electronic excitonic splitting *Δ*_exc_. From the above model and assuming *V*_AB_ to be small, the energy levels are obtained by first-order perturbation theory as:^12^,^28^

7 *E*_v′_^±^ = *E*_v′_ ± *V*_AB_〈*χ̃*Av′|*χ*A0〉〈〉〈*χ̃*Bv′|*χ*B0〉

Since the vibrational overlap integrals for monomers A and B are identical, the spacing between a given pair of excitonically split vibronic bands is

8 *Δ*_vibron_ = 2*V*_AB_||〈*χ̃*_v′_|*χ*_0_〉||^2^

This means that the purely electronic excitonic splitting 2*V*_AB_ is reduced by the (dimensionless) Franck–Condon factor (FCF), which is always smaller than unity. When specializing to the splitting between the S_1_/S_2_ state electronic origins, we obtain the quenching factor *Γ*9
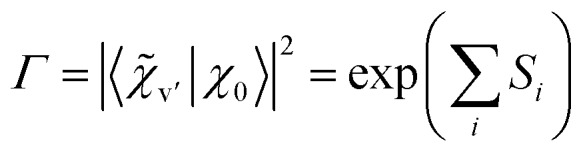
where *S*_*i*_ = FCF(*i*_0_^1^)/FCF(000) is the (dimensionless) Huang–Rhys factor of the *i*-th vibrational coordinate. For the symmetric H-bonded dimers in Fig. 1 we have determined the quenching of the S_1_/S_2_ excitonic splitting both computationally (from calculations of the monomer excited state vibrational potentials) and experimentally (from the fluorescence emission spectra of the respective monomers). The *ab initio* calculated vibronic quenching factors *Γ*_calc_ of the dimers in Fig. 1 are listed in Table 3 and lie between *Γ* = 0.019–0.228. When comparing the quenched splittings *Γ*_calc_·*Δ*_vert_ to the experimental excitonic splittings *Δ*_exp_ for the five dimers, see column 5 of Table 3, this approach yields good estimates of the observed excitonic splittings.^26^,^28^ In fact, when correcting the *Δ*_vert_ with the experimental *Γ*_exp_ values deduced from the monomer experimental fluorescence emission spectra, the agreement with experiment is near-quantitative, reconciling theory and experiment.^28^,^30^,^32^

**Table 3:** Calculated excitonic splittings, *Δ*_vert_ and quenching factors *Γ*_calc_ (dimensionless) and the resulting vibronic splittings *Δ*_vibron_ compared to the experimental excitonic splittings *Δ*_exp_ of the H-bonded dimers in Fig. 1 (*Δ* values in cm^–1^)

	*Δ*_vert_	*Γ*_calc_	*Δ* calc vibron	*Δ*_exp_
(2-Pyridone)_2_	1125	0.019	21.4	43.5
(*o*-Cyanophenol)_2_	309	0.067	20.7	16.4
(2-Aminopyridine)_2_	416	0.102	42.2	11.5
(Benzonitrile)_2_	10	0.228	2.3	2.1
(Benzoic acid)_2_	11	0.189	2.1	0.9

## 6 Band intensities

### 6.1 S_1_/S_2_ origin band intensities

As discussed above, the A_g_ → A_g_ transition is forbidden in exactly centrosymmetric dimers, and for the dimers in Fig. 1 the S_0_ → S_1_ band is not observed. The excited state wave functions corresponding to the S_1_ and S_2_ states can be written as:^12^,^29^,^53^

10 *Ψ*^+^ = cos *α*·*φ*Aexc*φ*B0 + sin *α*·*φ*A0*φ*Bexc

11 *Ψ*^–^ = sin *α*·*φ*Aexc*φ*B0 – cos *α*·*φ*A0*φ*Bexc

where the angle *α* is specified by requiring that^12^,^53^

12 tan(2*α*) = |*Δ*_exc_|/(|*E*_A*B_ – *E*_AB*_|).

For the symmetric homodimers *E*_A*B_ and *E*_AB*_ are degenerate, giving *α* = π/4, and the coupled exciton states are completely delocalized over both monomers. For slightly asymmetric dimers such as the ^13^C-isotopomers, the excitation energy difference between the isotopically substituted monomer and the non-substituted all-^12^C-monomer results in a partial localization of the S_1_ and S_2_ excited state wave functions on A or B. Given the electronic oscillator strength of the monomer, *f*_el,mono_, the relative electronic oscillator strengths of the S_0_ → S_1_ and S_2_ dimer are^29^

13 *f*_el,dimer_^+^ = (1 + 2 cos *α*·sin *α*)*f*_el,mono_

14 *f*_el,dimer_^–^ = (1 – 2 cos *α*·sin *α*)*f*_el,mono_

**Fig. 5:**
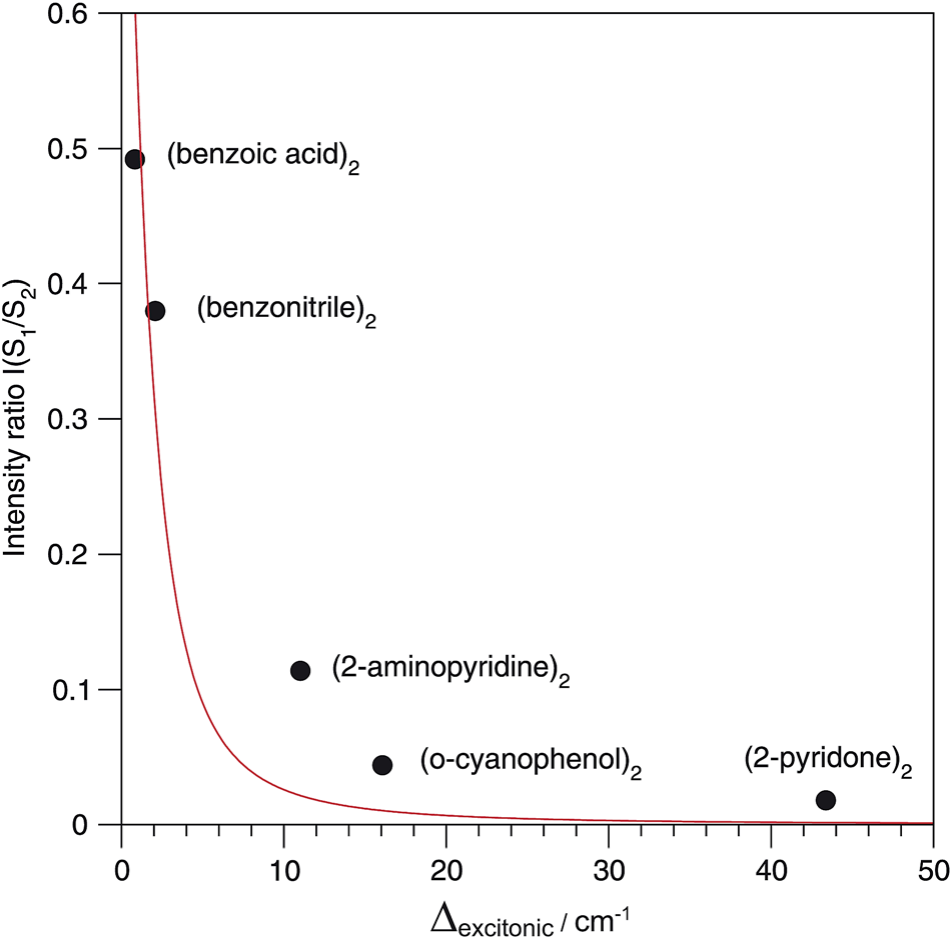
Dependence of the S_1_/S_2_ intensity ratio on the excitonic splitting in H-bonded symmetric molecular dimers. The excitonic splittings were determined from the directly observable splittings of the ^13^C isotopomers, assuming a ^12^C/^13^C isotopic shift *Δ*_iso_ of 3.3 cm^–1^ for all dimers. The theoretical dependence of the S_1_/S_2_ intensity ratio on *Δ*_exc_ is indicated in red.

The relative S_1_/S_2_ band intensity depends on the excitonic splitting *Δ*_exp_ and the isotopic shift between the origins of A and B, *Δ*_iso_ = *E*_A*_ – *E*_B*_. In Fig. 5, the intensity ratios *I*(S_2_)/*I*(S_1_) of the S_0_ → S_1_ and S_0_ → S_2_ origin bands of the ^13^C-isotopomers of the five dimers are plotted as a function of the excitonic splitting *Δ*_exp_. These experimental intensity ratios are compared to the ratio of oscillator strengths calculated with eqn (13) and (14). Using the same ^12^C/^13^C isotopic shift *Δ*_iso_ = 3.3 cm^–1^ (as determined experimentally for (benzoic acid)_2_ and (benzonitrile)_2_^28^,^30^) for all dimers, the intensity ratio is very nicely predicted by eqn (13) and (14). The deviation for (2-aminopyridine)_2_ is expected due to its non-planar *C*_2_-symmetric S_0_ state geometry, where the absence of inversion symmetry induces finite intensity of the S_0_ → S_1_ origin of the all-^12^C dimer.

### 6.2 Delocalization and exciton hopping in the excited state

For all the symmetric dimers studied, the symmetry lowering by ^12^C/^13^C isotope substitution results in the appearance of the S_0_ → S_1_ electronic origin. Its intensity relative to the S_0_ → S_2_ origin increases with decreasing splitting between the S_1_ and S_2_ origins, as the result of the decrease of the excitonic splitting *Δ*_exc_ relative to *Δ*_iso_, see Fig. 2(b). Given the excitonic splittings *Δ*_exc_ in Table 2, eqn (1) shows that the observed S_1_/S_2_ splitting *Δ*_obs_ is dominated by *Δ*_iso_ for the (benzonitrile)_2_ and (benzoic acid)_2_ dimers, which have small monomer S_0_ → S_1_ transition dipole moments. In this limit, the excitonic states become localized on the isotopically distinct A or B moieties, as is clearly visible in Fig. 3(d and e). In both cases, the S_1_ electronic 000 band is almost fully localized on the ^13^C-monomer while the S_2_ 000 band is localized on the all-^12^C-monomer. These observations confirm the interpretation of full delocalization of the dimer excited states over both chromophores in case of the symmetric dimers without isotope substitution.

In earlier spectroscopic studies of the symmetric dimers (benzoic acid)_2_,^36^,^38^ (benzonitrile)_2_,^37^,^54^ (*o*-cyanophenol)_2_,^39^,^40^,^55^ and (anthranilic acid)_2_,^41^ the authors repeatedly discussed whether the excited state is localized or delocalized. Based on the observation of non-totally symmetric vibrational bands in the supersonic jet electronic spectra,^39^,^40^,^55^ on the analysis of the excited-state IR NH-stretch bands of (anthranilic acid)_2_,^41^ and on the slightly asymmetric structure of (benzoic acid)_2_^36^,^38^ and (benzonitrile)_2_,^37^,^54^ derived from the rotationally resolved vibronic laser spectra, the electronic excitation was postulated to be localized on one of the monomers. This argument is unconvincing, since one then expects the appearance of a second electronic origin with similar intensity that is localized on the other moiety. However, no such second electronic origin was identified in any of the discussed complexes.^36^–^41^,^55^ In contrast, our recent work has revealed the appearance of the second 000 band of (benzoic acid)_2_, (benzonitrile)_2_ and (*o*-cyanophenol)_2_ if the *C*_2h_ symmetry is lowered to *C*_S_ by isotopic substitution, with concomitant localization of the electronic excitations.^29^,^30^,^33^

**Fig. 6:**
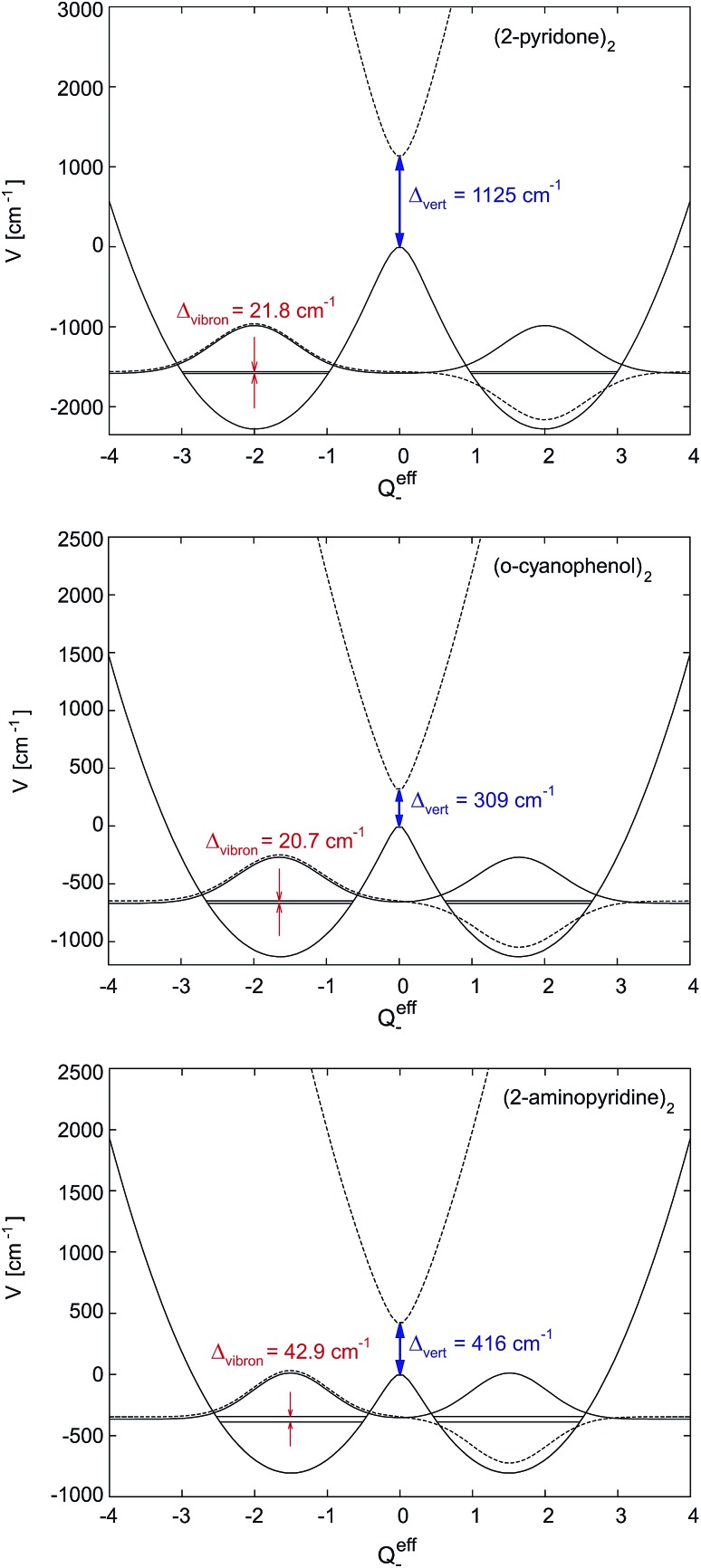
1D-cuts of the first two excited adiabatic potential energy surfaces along the effective mode for (2-pyridone)_2_, (*o*-cyanophenol)_2_ and (2-aminopyridine)_2_. The line type of the vibronic wave functions (schematic drawing) is the same as that of the corresponding potential energy curve, its zero is chosen to match its vibronic energy. The excitation energy splitting at the ground state equilibrium geometry (*Q*eff– = 0) equals the electronic excitonic splitting *Δ*_vert_.

As discussed in Section 5.2, the appearance of non-totally symmetric vibronic excitations is fully compatible with delocalized excitonic states, since the vibronic coupling in weak-coupling systems results in the appearance of additional bands, as shown in Section 5. The asymmetric dimer geometry that is implied by the two S_1_-state minima along the asymmetric vibrational mode *Q*_–_ for the weak-coupling case in Fig. 4(b) do not contradict the interpretation of the excitonic excitation as being delocalized. Instead, considering the total effect of vibronic coupling to the totally symmetric monomer modes, this is expected, since the delocalized excited state wave functions of both the S_1_ and S_2_ states have the highest probability density close to the two equivalent minima of the double minimum potential (note that the levels corresponding to the vibronic S_1_ and S_2_ origins are both located in the lower (double-minimum) potential in Fig. 6). Thus the most probable geometry of (benzoic acid)_2_ and (benzonitrile)_2_ in their S_1_ and S_2_ excited states should be slightly asymmetric, in agreement with the structures determined by high-resolution laser measurements of the 000 bands.^36^–^38^,^54^

In a semiclassical picture, the excitation can be considered to be hopping between the A and B chromophores, with a resonance transfer rate^12^

15 *k*_AB_ = 4|sin(2*α*)||*V*_AB_|/*h*

where the angle 2*α* = tan^–1^(|*V*_AB_|/|*E*_A*B_ – *E*_AB*_|). For the symmetric complexes, with *α* = π/4, the time constant depends only on the excitonic coupling element *V*_AB_ = *Δ*_exc_/2. We emphasize that the observation of real-time dynamics along these lines requires a coherent excitation of the split 000 bands with sufficiently short (sub-ps) excitation laser pulses. Since both band origins must have nonzero oscillator strength, such experiments are feasible for the symmetry-broken systems (2-aminopyridine)_2_ and the ^13^C-isotopomers, but not for the *C*_2h_-symmetric ^12^C-isotopomers. Time-resolved observation of excitonic splittings – while experimentally possible – is not described here, and our discussion is more of a heuristic nature.

For the ^13^C- and D-substituted dimers the difference in excitation energy between the two inequivalent chromophores *E*_A*B_ – *E*_AB*_ results in an increase of the hopping time. The hopping times for the symmetric (benzoic acid)_2_ and (benzonitrile)_2_ have been determined as *t*_exc_ = *k*_AB_^–1^ = 17.7 and 8.0 ps.^29^,^30^ This means that the symmetric homodimers are not only electronically symmetric, but that the vibrational asymmetry along the antisymmetric coordinates is averaged out on this timescale. The hopping times for the corresponding ^13^C-(benzoic acid)_2_ and ^13^C-(benzonitrile)_2_ increase significantly to 124 ps and 15 ps, respectively.

## 7 Adiabatic description and effective mode approximation

### 7.1 One-dimensional effective mode description

The above interpretation (Sec. 5.1) of the quenched exciton splitting is based on perturbation theory using the locally excited states of the monomers as zero-order states (they are degenerate for symmetry-equivalent monomers, as is always assumed in this section). These electronic states are an example of diabatic states as they are not eigenstates of the electronic Hamiltonian of the dimer. As an alternative, Kopec *et al.* have formulated an approach explaining the quenching of the excitonic splitting in molecular dimers based on adiabatic electronic states and potential energy surfaces (PES).^32^ The adiabatic PES are defined as the eigenvalues of the fixed-nuclei part of the Hamiltonian (6), *i.e.* dropping the nuclear kinetic energy part, and are best written as functions of the symmetric and antisymmetric linear combinations of the monomeric coordinates *Q*_A_ and *Q*_B_. In dimensionless form they read16
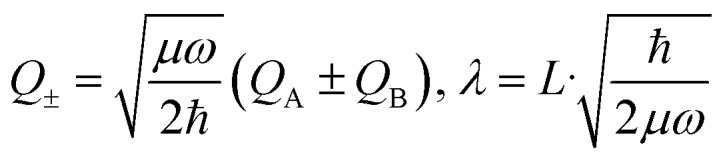
leading to the following expression for the adiabatic PES:17
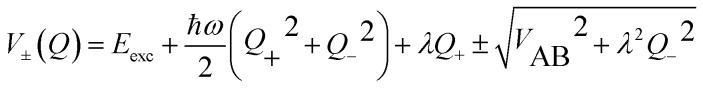



For the relevant case of weak excitonic coupling the lower surface *V*_–_ has a double-minimum shape (see below) with a stabilization energy *E*_stab_ (or, equivalently, energy barrier at *Q* = 0 separating the two equivalent distorted minima) of18
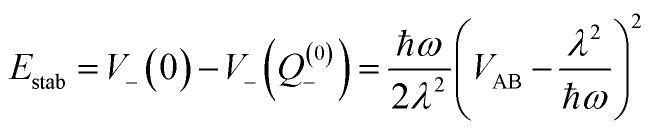



The displacement along the asymmetric mode at the distorted minima reads19
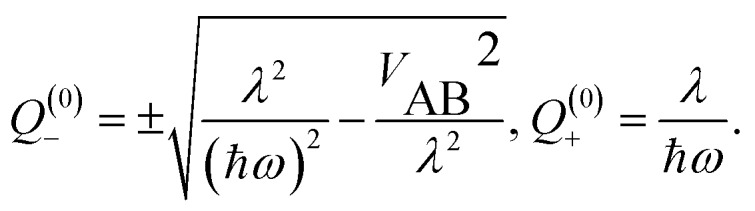



The above expressions can be readily generalized for the ubiquitous case of several vibronically active modes.^32^ Rather than giving lengthy equations we just note that the following quantities appearing in the above expressions for the stabilization energy and asymmetric distortion are to be replaced as follows:20
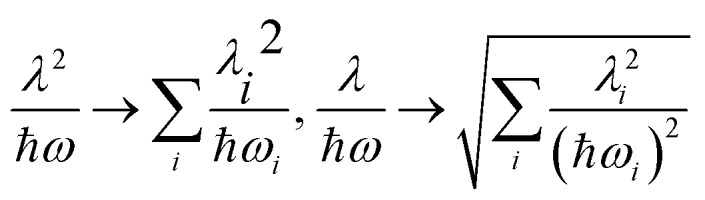



This leads to an elegant construction of an *effective* antisymmetric vibrational mode, which allows to visualize the total effect of all relevant modes in a single dimension. The latter is defined by requiring that the distortion and stabilization energy for this single mode correctly reproduce that of the full multi-mode system as given by eqn 18–20. It leads to frequency *Ω* and coupling constant *Λ* of the effective mode defined as in eqn (20) where the arrows are replaced by equal signs.

### 7.2 Nonadiabatic tunneling interpretation

Fig. 6 shows the symmetric-dimer S_1_- and S_2_-state effective mode potentials, with the quenched vibronic splitting *Δ*_vibron_ and the purely electronic splitting *Δ*_vert_ for two representative cases. It illustrates the above discussed discrepancy between the *ab initio* calculated and the experimentally observed excitonic splittings. Indeed, the *ab initio* methods yield rather accurate results for the purely electronic S_1_–S_2_ energy gap but this splitting is *not observable* in any excitonic molecular dimer. The actual vibronic S_1_ and S_2_ origins correspond to the energy levels indicated, on which, due to weak coupling, two strongly overlapping vibrational band structures would build up.

The quenched vibronic energy splittings given in the figure have been obtained by numerically diagonalizing the FG Hamiltonian with the effective mode parameters obtained as described above and the same underlying multi-mode coupling constants as used in the perturbation theoretical approach of Sec. 5.1. The effective mode results of Fig. 6 can thus be directly compared with the corresponding entries in the column 4 of Table 3. The excellent agreement between the two approaches should be noted and mutually confirms the reliability of the different approximations.

The double minimum-shape of the lower adiabatic PES *V*_–_ suggests an interpretation of the quenched excitonic splitting as originating from quantum tunneling on *V*_–_. It should however be born in mind that the calculated splitting results from a coupled-surface vibronic computation and includes the influence of the upper PES *V*_+_ on the tunneling motion. To explicitly reveal this, we have recomputed the effective-mode excitonic splitting as pure tunneling splitting on *V*_–_ by suppressing the nonadiabatic coupling to *V*_+_.^32^ This results in splittings about 3–6 times larger than those given in Fig. 6. The quenched splittings are thus due to *nonadiabatic* tunneling between the two conformations where the excitation rests primarily on one of the two monomers. We emphasize that the energy gap of the interacting states is always kept fixed at the *ab initio* value *Δ*_vert_ indicated, for example, in Fig. 6. Therefore the nonadiabatic coupling effects are not subject to the ambiguity discussed in ref. 56. Nevertheless, in a genuine multimode treatment some deviations may arise.

Recent methodological developments in our groups^57^ have given a very simple closed-form expression which accurately reproduces the exciton splitting due to nonadiabatic tunneling. To this end the perturbation theoretical approach of Sec. 5.2 is applied to the localized ground state vibrational wave functions of the left and right potential wells of *V*_–_(*Q*eff–) as depicted in Fig. 6. The latter wells, and the ground state vibrational wave functions, are taken to be harmonic. Due to the very construction of the effective mode (see end of Sec. 7.1), the stabilization energy as well as total distortion of the many-mode problem is captured exactly by the effective mode. In the limit of vanishing excitonic splitting *Δ*_vert_ the vibrational overlap, and hence the vibrational quenching, of Sec. 5.2 are thus exactly recovered. For small finite splitting *Δ*_vert_ the vibrational ground state wave functions can be easily computed in the harmonic limit. The only effect of nonzero *Δ*_vert_(=2*V*_AB_) is a small decrease of *Q*(0)– in eqn (19) compared to the case *V*_AB_ = 0. The vibrational overlap is thus slightly increased, the quenching slightly decreased and the quenched excitonic splitting again slightly increased in this approach compared to the earlier version of Sec. 5.2. In practice the differences are very minor, amounting to only 1 cm^–1^ or less, and the quenched excitonic splittings *Δ*_vibron_ lie in the same range as in Table 3 and Fig. 6. Thus, the combined effects of the effective mode and nonadiabatic tunneling are incorporated in this modified perturbation-theoretical approach.

## 8 Conclusions

The investigation of the rigid *C*_2h_ or *C*_2_ symmetric H-bonded dimers (2-pyridone)_2_, (*o*-cyanophenol)_2_, (2-aminopyridine)_2_, (benzonitrile)_2_ and (benzoic acid)_2_ by species- and isotope-selective laser spectroscopic methods have revealed that the symmetric dimers exhibit only a single vibronic band system, which is typically the S_0_ → S_2_ excitation, due to symmetry selection rules. However, even minimal symmetry breaking by replacing a single ^12^C atom by a ^13^C atom reveals the existence of the close-lying S_0_ → S_1_ band system. The energy difference between the S_1_ and S_2_ 000 bands corresponds to the excitonic splitting in these dimers.

The observed excitonic splittings *Δ*_exp_ are typically 5–25 times smaller than the energy gaps between the S_1_ and S_2_ states that are calculated vertically at the ground-state minimum geometry. The large difference between the calculated and experimental splittings results from vibronic coupling between the two degenerate electronic states, and can be considered a consequence of the Born–Oppenheimer approximation that is inherent to the *ab initio* calculations.

The vibronic coupling model introduced by Witkowski^15^ in 1960 and subsequently extended and solved by Fulton and Gouterman^18^,^19^ includes two electronic states that are coupled to a pair of vibrations of the dimer (one per monomer). The FG model can be adapted to reproduce the observed band structure and splitting for one pair of vibrations at a time, although its application to intermonomer degrees of freedom was not anticipated by FG and is purely phenomenological.^26^,^51^ The development of a one-dimensional effective mode vibronic coupling description^32^ projects the multidimensional couplings of all vibrational modes of the dimers onto a single antisymmetric vibrational coordinate, thereby giving valuable insights into the S_1_/S_2_ potential shapes, vibronic quenching behavior and interpretation of the excited-state geometry of the dimers.^28^–^30^,^32^

The experimental excitonic splittings in these dimers are then obtained by correcting the large excitonic splittings predicted by the Born–Oppenheimer-based *ab initio* calculations by the vibronic quenching factor *Γ*, which can be obtained from the experimental or calculated S_0_ ↔ S_1_ spectra of the respective monomer moieties. From a perturbation-theoretical point of view, *Γ* can be viewed as arising from the product of the excited-state vibrational displacements (Huang–Rhys factors) along the optically active vibrations of the monomer, thereby fragmenting the *ab initio* calculated electronic oscillator strength *f*_el_ into the much smaller vibronic oscillator strengths of the vibronic fundamental excitations, *f*_vibron_ ≪ *f*_el_.^27^,^28^,^32^ The smaller *f*_vibron_ give rise to proportionally smaller excitonic splittings between the respective pairs of vibronic transitions of the dimer.^27^ In the five dimers discussed here, *Γ* = 0.03–0.25.

Recent multimode vibronic coupling calculations were able to reproduce the observed band patterns in the spectrum of (*o*-cyanophenol)_2_,^33^ and explain the excitonic splittings not only of the intramolecular, but also of the *intermolecular* vibrations. Related methods for the interpretation of vibronic coupling in a single molecule containing two weakly coupled chromophores have been developed by Slipchenko and co-workers,^31^,^34^ and have been applied to the vibronic spectra of diphenylmethane and several of its derivatives.^58^–^60^

Future challenges will involve the exploration of excitonic couplings in dimers that are more strongly asymmetrized than by ^12^C/^13^C-substitution. Examples are asymmetrization by H/D isotopic exchange or by chemically attaching a methyl group to one of the chromophores.^51^ More extreme cases are excitonic dimers that do not fulfill the requirement of inversion symmetry, such as the paradigmatic T-shaped aromatic dimers (benzene)_2_,^61^–^65^ and (naphthalene)_2_,^66^ in which the A and B monomers are symmetry-inequivalent.
